# Genotyping Test with Clinical Factors: Better Management of Acute Postoperative Pain?

**DOI:** 10.3390/ijms16036298

**Published:** 2015-03-19

**Authors:** Aline Hajj, Katell Peoc’h, Jean-Louis Laplanche, Hicham Jabbour, Nicole Naccache, Hicham Abou Zeid, Patricia Yazbeck, Lydia Rabbaa Khabbaz

**Affiliations:** 1 Laboratoire de Pharmacologie, Pharmacie Clinique et Contrôle de Qualité des Médicaments, Faculté de Pharmacie, Saint-Joseph University of Beirut, B.P. 11-5076-Riad El Solh, Beirut 1107 2180, Lebanon; E-Mail: lydia.khabbaz@usj.edu.lb; 2 Université Paris Descartes, Unité INSERM UMR-S 1144, Paris F-75006, France; E-Mails: katell.peoch@bjn.aphp.fr (K.P.); jean-louis.laplanche@inserm.fr (J.-L.L.); 3 Department of Anesthesia and Critical Care, Hôtel-Dieu de France Hospital-Saint-Joseph University of Beirut, B.P. 16-6830, Beirut 1100 2160, Lebanon; E-Mails: hicham.jabbour@usj.edu.lb (H.J.); nicole.naccache@usj.edu.lb (N.N.); hicham.abouzeid1@usj.edu.lb (H.A.Z.); patricia.yazbeck@usj.edu.lb (P.Y.)

**Keywords:** morphine, polymorphism, *OPRM1*, *ABCB1*, pain, pharmacogenetics

## Abstract

Individualization of acute postoperative pain treatment on an evidence-based decision process is a major health concern. The aim of this study is to investigate the influence of genetic and non-genetic factors on the variability of response to morphine in acute postoperative pain. A group of nighty-five patients undergoing major surgery were included prospectively. At 24 h, a logistic regression model was carried out to determine the factors associated with morphine doses given by a Patient Controlled Analgesia device. The dose of morphine was associated with age (*p* = 0.011), patient weight (*p* = 0.025) and the duration of operation (*p* = 0.030). This dose decreased with patient’s age and duration of operation and increased with patient’s weight. *OPRM1* and *ABCB1* polymorphisms were significantly associated with administered dose of morphine (*p* = 0.038 and 0.012 respectively). Patients with at least one G allele for c.118A>G *OPRM1* polymorphism (AG/GG) needed 4 times the dose of morphine of AA patients. Additionally, patients with *ABCB1* CT and CC genotypes for c.3435C>T polymorphism were 5.6 to 7.1 times more prone to receive higher dose of morphine than TT patients. Our preliminary results support the evidence that *OPRM1*/*ABCB1* genotypes along with age, weight and duration of operation have an impact on morphine consumption for acute postoperative pain treatment.

## 1. Introduction

Management of acute postoperative pain is a major challenge. Studies suggest that only 25% of postoperative patients receive appropriate analgesic treatment [1,2]. Pain control in this context remains an under-managed healthcare issue despite the introduction of clinical standards, guidelines, and educational efforts. According to international guidelines, opioids are the mainstay of analgesic therapy; they are the most commonly used drugs to control moderate to severe acute pain [3]. Opioids have a narrow therapeutic index and are characterized by a large inter-individual variation in both response and efficacy.

Patient controlled analgesia (PCA) is a frequently used system to manage post-operative pain. It relies on the use of a pump that allows patients to self-administer morphine according to their subjective evaluation of pain. Consequently, the administered morphine doses vary greatly among patients.

Many studies have investigated the association between genetic and non-genetic factors and the variability of response to opioids [4].

Genetic factors are thought to be responsible for approximately 12% to 60% of response variability in opioid treatment, as evaluated by twin studies [5].

Many genes have been studied to identify pharmacogenetic markers in morphine treatment, including genes implied in pharmacodynamics (such as *OPRM1*) and pharmacokinetics (such as *ABCB1*) that can modulate nociception and analgesic dose requirement.

*OPRM1* encodes for the Mu opioid receptor (MOR). Studies on mice with targeted inactivation for *Oprm1* established this receptor as essential for morphine analgesia, physical dependence, and reward process [6]. More than 100 single nucleotide polymorphisms (SNPs) have been described in *OPRM1* [7]. The common polymorphism c.118A>G (rs1799971), which has been extensively studied, leads to an asparagine to aspartate substitution (p.Asn40Asp), with an allelic frequency varying from 2% to 50% according to ethnic groups [8]. This polymorphism abolishes a putative N-linked glycosylation site in the *N*-terminal domain of the receptor associated with a modification of responses to opiates [9]. Indeed, the variant protein exhibits three times greater binding affinity for the endopeptide β-endorphin, whereas binding to morphine, methadone and naloxone are unaffected *in vitro* [9]. c.118A>G is also associated with MOR expression, the variant being associated with a decrease in both mRNA expression and translation into functional protein [10].

In addition, numerous studies have shown that some opioids, in particular morphine, are substrates for the P-glycoprotein (P-gp) [11,12,13]. The P-gp is a transmembrane efflux transporter belonging to the family of ATP binding cassette (ABC) transporters. P-gp is encoded in humans by *ABCB1* (former *MDR1* for multi-drug resistance protein). The c.3435C>T (rs1045642) variant is a result of a C-to-T substitution at nucleotide 3435 and has been associated with a reduced expression of duodenal P-gp in homozygous TT patients [14]. It has also been associated with a 1.5- to 2-fold reduction in mRNA levels and/or a reduction in protein expression in some tissues [15].

As for non-genetic factors, numerous studies have shown that age, gender, hepatic or renal failure, anxiety, and duration of surgery are predictive factors of morphine requirements in the post-operative period [16,17,18].

The aim of this study was to investigate the association between genetic or non-genetic factors and morphine requirements using PCA in the 24 h post-operative period. Therefore, we evaluated the allelic frequencies of two SNPs (rs179997, rs1045642) in a Lebanese population of patients undergoing orthopedic or major abdominal surgeries. We then assessed the relationship between these genetic and demographic/clinical factors on one hand, and pain perception and morphine doses and side effects on the other hand.

## 2. Results and Discussion

### 2.1. Patients Population

A total of 100 patients (mean age 51 years old, 40% male and 60% female) were enrolled in this study. Out of these patients, five were then excluded: three patients having received remifentanyl during surgery and one having refused the installation of PCA. In addition, in one patient, a desaturation with hypovolemia and postoperative bleeding occurred 2 h postoperatively. The PCA was stopped and the patient was sent to the intensive care unit. Consequently, only 95 patients completed the study. The main clinical and demographic characteristics of patients are presented in Table 1.

**Table 1:** Characteristics of the patients.

Characteristics of the Subjects (*N* = 95)	*N*
Women	57 (60.0%)
Type of surgery	
Urogynaecology N (%)	45 (47.4%)
Orthopaedic N (%)	38 (40.0%)
Gastroenterology N (%)	12 (12.6%)
	**Mean ± SD**
Age (years)	51.1 ± 14.0
Weight (Kg)	75.6 ± 14.7
Height (cm)	166.8 ± 7.8
Creatinine clearance (mL/min)	67.6 ± 22.1
Dose of Fentanyl (microg)	280.0 ± 92.2
Urogynaecology	268.0 ± 81.5
Orthopaedic	298.5 ± 98.1
Gastroenterology	270.8 ± 109.7
Duration of operation (min)	216.7 ± 119.1
Urogynaecology	201.2 ± 108.4
Orthopaedic	213.9 ± 117.1
Gastroenterology	283.3 ± 149.5
VAS	
24 h at rest	0.98 ± 1.4
48 h at rest	0.59 ± 1.2
24 h on movement	1.95 ± 1.7
48 h on movement	1.29 ± 1.4

Patients were admitted for urogynaecology (47.4%), orthopaedic (40%) or gastroenterology (12.6%) surgeries.

The mean pain scores evaluated by the visual analog scale (VAS) at rest and on movement were relatively low at 24 h (VAS at rest 0.98 ± 1.4; VAS on movement 1.95 ± 1.7). Neither the VAS at rest (*p*_24_ = 0.53) nor the VAS on movement (*p*_24_ = 0.22) was significantly different between the various surgery groups.

Morphine doses administered by PCA were highly variable within the population: 4 to 227 mg per 24 h. The median doses (±SD) were 40.5 ± 34.9 mg.

### 2.2. Genotype and Allele Distribution

Results of genotyping and allele distribution are summarized in Table 2. Concerning *OPRM1* c.118A>G, the allelic frequencies were 0.89 for 118A and 0.11 for 118G. For *ABCB1* c.3435C>T, allelic frequencies were 0.55 for 3435C and 0.45 for 3435T (*n* = 192 chromosomes). The population was in Hardy-Weinberg equilibrium for both SNPs.

**Table 2:** Genotype and allele frequencies of *OPRM1* and *ABCB1* variants in our population. Comparison with previously published data.

Gene	dbSNP	Genotype Frequencies ^a^			Allelic Frequencies ^a^		*p* ^b^
***OPRM1***	rs1799971	AA	AG	GG	A	G	
	Lebanese patients *n* = 96 (Current study)	76 (79.2)	18 (18.8)	2 (2.1)	0.89	0.11	–
	European HapMap *n* = 113	80 (70.8)	31 (27.4)	2 (1.8)	0.84	0.16	0.336
	Japanese HapMap *n* = 86	29 (33.7)	34 (39.5)	23 (26.7)	0.53	0.47	0.0001 *
	Chinese HapMap *n* = 43	18 (41.9)	19 (44.2)	6 (14)	0.64	0.36	0.0001 *
	Sub-Saharan African HapMap *n* = 60	60 (100)	0 (0)	0 (0)	1	0	0.0008 *
***ABCB1***	rs1045642	CC	CT	TT	C	T	
	Lebanese patients *n* = 96 (Current study)	34 (35.4)	38 (39.6)	24 (25)	0.55	0.44	–
	European HapMap *n* = 113	17 (15)	63 (55.8)	33 (29.2)	0.43	0.57	0.0025 *
	Japanese HapMap *n* = 86	22 (25.6)	49 (57)	15 (17.4)	0.54	0.46	0.063
	Chinese HapMap *n* = 42	16 (38.1)	17 (40.5)	9 (21.4)	0.58	0.42	0.895
	Sub-Saharan African HapMap *n* = 113	89 (78.8)	23 (20.4)	1 (0.8)	0.89	0.11	0.0001 *

^a^ Value represents the number of patients with percentage shown in parenthesis; ^b^ *p* values are obtained using χ^2^ test between the number of patients of each genotype compared to our study [19,20]; * Statistically significant result.

### 2.3. Variables Associated with Morphine Doses

In order to explore the variables associated with morphine doses, a univariate analysis was conducted after dichotomization of the doses of morphine using the 50th percentile. The comparison of the distribution and mean values of patient socio-demographic and genetic characteristics as well as surgery parameters are presented in Table 3.

**Table 3:** Comparisons of patients’ distribution and mean values of characteristics between groups of subjects differing in morphine dose at 24 h.

Characteristics of the Subjects	Dose of Morphine at 24 h (mg)		
	Dose ≤ 41 mg (*n* = 48)	Dose > 41 mg (*n* = 47)	*p*
Age (years) Mean ± SD	54.6 ± 13.8	47.4 ± 13.4	0.012 *
Female N (%)	29 (60.4%)	28 (59.6%)	1.000
Weight (Kg) Mean ± SD	72.6 ± 13.1	78.6 ± 15.8	**0.045**
Height (cm) Mean ± SD	166.2 ± 7.9	167.4 ± 7.8	0.440
Type of surgery			
Urogynecology N (%)	27 (56.2%)	18 (38.3%)	**0.176**
Orthopedic N (%)	15 (31.2%)	23 (48.9%)	
Gastroenterology N (%)	6 (12.5%)	6 (12.8%)	
Dose of Fentanyl (microg) Mean ± SD	270.1 ± 101.3	290.7 ± 81.1	0.295
Duration of operation (mn)/Mean ± SD	239.6 ± 125.5	193.3 ± 108.7	**0.058**
*ABCB1* c.3435C>T			
CC	12 (25.0%)	21 (44.7%)	**0.004**
CT	17 (35.4%)	21 (44.7%)	
TT	19 (39.6%)	5 (10.6%)	
*OPRM1* c.118A>G			
AA	43 (89.6%)	33 (70.2%)	**0.018**
AG and GG	5 (10.4%)	14 (29.8%)	

* The numbers in bold represent the explanatory variables that showed associations to the doses of morphine with *p* < 0.25 in the univariate analyses.

The explanatory variables that showed associations to the doses of morphine with *p* < 0.25 in this univariate analyses (in bold in Table 3) were introduced in the multivariate model. The results of the binary logistic regression for morphine dose at 24 h are presented in Table 4.

The 24 h morphine dose was associated with age (*p* = 0.011), weight (*p* = 0.025) and the duration of operation (*p* = 0.030) (Table 4). The mean dose of morphine decreased with patient age as well as with the duration of operation and increased with patient weight.

**Figure 1:**
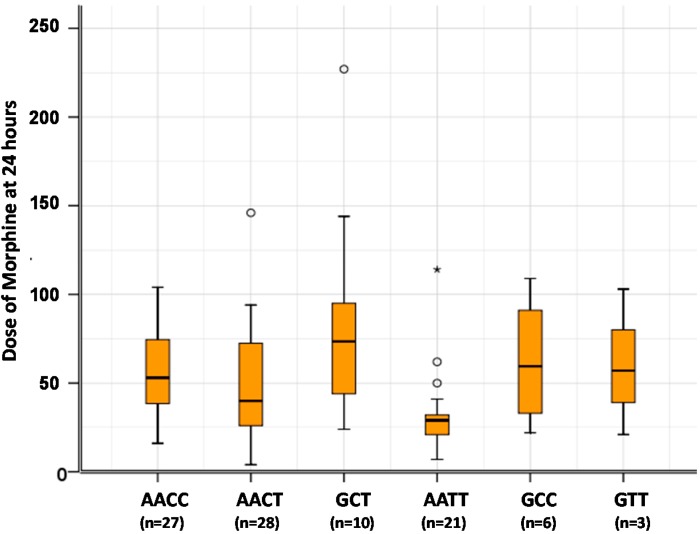
Doses of morphine at 24 h based on genotype distribution for both *OPRM1* and *ABCB1* SNPs. Morphine dose requirements according to the genotypes for the two SNPs *OPRM1* c.118A>G and *ABCB1* c.3435C>T. Due to the small number of 118GG patients, the results are grouped by G allele (the patients carrying at least one G allele). o Outliers or doses values that extend more than 1.5 box-lengths from the edge of the box; * Extreme values or doses values that extend 3 box-lengths from the edge of the box.

*OPRM1* and *ABCB1* SNPs were significantly associated with morphine doses at 24 h (*p* = 0.038 and 0.012 respectively). Patients with at least one G allele for c.118A>G *OPRM1* (AG/GG) needed 4 times the dose of morphine of AA patients. Additionally, patients with *ABCB1* CT and *ABCB1* CC genotypes were 5.6 to 7.1 times more likely to receive a higher dose of morphine than *ABCB1* TT patients (Figure 1).

**Table 4:** Binary logistic regression for morphine dose at 24 h.

Characteristics of the Subjects	B ^a^	S.E. ^b^	Sig. ^c^	OR ^d^	95.0% CI for OR	
					Lower	Upper
Age	−0.048	0.019	**0.011** ^f^	0.953	0.918	0.989
Weight	0.042	0.019	**0.025**	1.042	1.005	1.081
Type of surgery	−0.114	0.367	0.756	0.892	0.434	1.833
Duration of operation	−0.005	0.002	**0.030**	0.995	0.990	0.999
*ABCB1* (TT Reference ^e^)			**0.012**			
*ABCB1* (CC)	1.954	0.683	0.004	7.060	1.850	26.943
*ABCB1* (CT)	1.719	0.682	0.012	5.579	1.465	21.236
*OPRM1* (AA Reference ^e^)						
*OPRM1* groups (AG/GG)	1.394	0.671	**0.038**	4.031	1.083	15.013

This is a table of multivariate analyses where all the confounding factors were included in the model in order to study the adjusted association of each explanatory independent variable with the dose of morphine. a—B is a logistic regression coefficient; b—S.E. is the standard error; c—Sig. is the significance level or the *p*-value; d—The odds ratio or OR is calculated according to Exp(B). The ratio of the coefficient (B) to its standard error, squared, equals the Wald statistic. If the significance level of the Wald statistic is less than 0.05 then the parameter is useful to the model. The logistic regression coefficient (B) is convenient for testing the usefulness of explanatory independent variable, however Exp(B) is easier to interpret. Exp(B) represents the ratio-change in the odds of the event of interest for a one-unit change in the predictor; e—To calculate the odds-ratio (OR), TT and AA were taken as reference as the patients with those genotypes required the lowest morphine doses; f—The numbers in bold represent the explanatory variables that showed associations to the doses of morphine with *p* < 0.05 in the univariate analyses.

### 2.4. Association to Side Effects and Visual Analog Scale (VAS) Scores

Side effects reported postoperatively were sedation and nausea/vomiting. However, the overall incidence of these adverse events remained low; only 11 patients out of 95 had sedation (13.2%; mean sedation_24_ score 0.13 (0–2)) and 14 patients had at least one episode of nausea/vomiting in the first post-operative 24 h (14.7%; mean nausea_24_ score 0.17 (0–2)).

None of the studied factors (SNPs, age, weight, duration of the operation) were associated with sedation nor nausea/vomiting scores at 24 h (Table S1–S9). However, an association was found between the duration of the operation and the VAS scores at 24 h (*p* = 0.033). No other association was found with the VAS scores at rest or on movement (Table S10–S12).

### 2.5. Discussion

Postoperative pain management remains a challenge for clinicians due to unpredictable patient’s response to opioid therapy. Morphine is the most widely used drug for postoperative pain treatment. Inter-individual variability in response to morphine is a significant challenge in the management of pain. A PCA morphine administration usually guarantees an optimal analgesia. However, some studies have shown that this technique is also associated with a risk of complications, even serious ones such as respiratory depression which was estimated at 0.25%–7% [21,22,23].

Indeed, some errors have been reported such as incorrect programming of the pump, wrong understanding or reproduction of the physician’s prescription and finally errors in monitoring and system malfunction leading to overdoses. In addition, some patients with psychomotor delay or suffering from psychiatric disorders, non-cooperative patients, and in particular elderly ones may be unable to adopt the technique or even understand its concept [16].

This study was conducted in order to evaluate the added value of determining some genetic and non-genetic factors in optimizing postoperative pain treatment. Rational doses of standard (morphine) or minor analgesics treatment might be sufficient for patients requiring low doses, whereas patients requiring high doses of morphine would be ideal candidates for a PCA-morphine based on a “multimodal” analgesia, combined with loco-regional or peripheral nerve blocks analgesia techniques.

In this study, patient age and weight were significant predictors of morphine consumption during the 24 h after surgery.

Elderly patients appeared to be more sensitive to the analgesic effect of morphine than younger patients. The association between increased patient’s age and reduction of morphine requirement for postoperative analgesia has been previously published [17,24,25]. Elderly patients have an altered distribution, metabolism, and elimination of morphine, which can explain their reduced need for morphine to achieve pain relief [17].

As for weight, patients with higher weight seem to have higher postoperative morphine consumption. Whereas the anesthetic opioid doses are “partly” adapted to the patient’s weight, the postoperative dose of morphine is mainly based on the level of pain and initial titration dose. However, our current observation is probably related to a higher volume of distribution.

A significant influence of surgical factors (duration of operation) on both VAS scores and morphine doses was found. Hence, the longer the duration of the operation, the more it was associated with high VAS scores but lower morphine doses. This result is not in agreement with previous work that reported an increase in postoperative opioid requirements with increased post-operative pain levels [18].

Genetic factors appear to influence both sensitivity to pain, and analgesic requirement for effective pain control.

The Lebanese population is an admixture of Ancient Phoenician pooled with Arabian and Western European lineages [26]. Indeed, allelic frequencies in our population were similar to either those described in Caucasian populations (European HapMap control population, *n* = 113; Table 2) for *OPRM1* c.118A>G or to those of Asian populations for *ABCB1* c.3435C>T (Chinese HapMap).

Previous studies have produced inconsistent results regarding the association of *OPRM1* c.118A>G and *ABCB1* c.3435C>T polymorphisms with morphine response [4].

In our study, patients with at least one 118G allele (AG and GG) for *OPRM1* received significantly higher doses of morphine than AA patients and those with at least one 3435C allele (CC or CT) for *ABCB1* received significantly higher doses of morphine than TT patients. Hence, 118AA and 3435TT patients may be considered as “good responders” to morphine.

Indeed, as previously described the c.118A>G SNP in *OPRM1* indicates a change from an asparagine to an aspartic acid residue in amino acid position 40 (p.N40D). Studies have shown that 118G allele expression in stably transfected cells results in a reduced expression of MOR at the cell surface due to both transcriptional and post-transcriptional effects [10,27], suggesting that this allele may be associated in patients with a reduced expression of the receptor leading to a reduced activation of the transduction pathway. This SNP has been reported to be associated with an increased binding affinity for the endopeptide β-endorphin *in vitro* [9] and reduced potency of morphine-6-glucuronide (M6G) [28]. However, it does not seem to affect the binding affinity of small molecules such as morphine [29]. This is probably the reason why a homozygous carrier of the mutant 118G allele of *OPRM1* needs larger doses of morphine to compete for opioid receptors. Hence, in this patient, MOR with an aspartate at residue 40 (corresponding to G-allele) would control analgesia less efficiently.

Numerous studies have evaluated the association of *OPRM1* c.118A>G and the doses required for pain relief (for review [4,30]). As reported in our study, most of the studies have demonstrated that 118GG patients or carriers of at least one 118G allele required higher morphine doses for post-operative pain relief [25,31,32,33].

Concerning *ABCB1* c.3435C>T, our results are in agreement with previously published data. Campa *et al.* (*n* = 145 Caucasians) showed that *ABCB1* TT patients were “good responders” compared to CC patients [34]. Other publications have shown that TT patients have lower morphine requirements than CC patients, as well as higher cerebrospinal fluid morphine concentrations [35,36,37]. Human P-gp is involved in the blood-brain barrier integrity and transports several drugs out of the tissues. We could hypothesize that 3435TT patients would have a lower P-gp expression and would therefore present less efflux of morphine at the intestine level and possibly at the blood brain barrier, and thus require lower doses of morphine to control their postoperative pain.

We did not detect any association between genetic or non-genetic factors with the occurrence of morphine side effects, probably due to the small sample size.

It is noteworthy to add that the results presented in this article might be considered as preliminary results. In fact, this is the first pharmacogenetic study conducted in Lebanon for pain management and it is very motivating to compare our results with other populations. Our aim was to determine the genetic and demographic factors associated with the dose of morphine in a homogeneous sample of Lebanese patients. We acknowledge however that it is a relatively small study for genetic associations and further studies are consequently needed on a larger sample to confirm and generalize our results on Lebanese patients as well as other populations.

Finally, we cannot exclude that other genetic effects (other SNPs in *OPRM1* and *ABCB1* and other genes such as those encoding glucuronidases ex. UGT2B7) contribute to the response variability to morphine.

## 3. Experimental Section

### 3.1. Study Design and Patients

In order to be included in the study, patients had to be Lebanese, older than 18 years and planning to undergo painful surgeries at Hôtel-Dieu de France Hospital (Saint-Joseph University of Beirut). Patients chosen to be enrolled in this study were candidates for orthopaedic or major abdominal surgeries requiring parenteral analgesia in the post-operative period and were supposed to experience the same levels of pain. Inclusion occurred after patients had provided a written informed consent. Patients were not eligible if their calculated creatinine clearance (estimated by the Cockcroft-Gault formula) was less than 40 mL/min. In addition, patients treated with opioid analgesics, corticosteroids or non-steroidal anti-inflammatory drugs in the preoperative period, or those taking on a regular basis co-analgesics such as benzodiazepines or antidepressants were also excluded because these drugs may alter pain perception.

This prospective study was conducted between 20 October 2009 and 6 June 2011 and was approved by the ethical committee of the hospital (Protocol N.256 bis, 2009; 09/JD/381, 9 September 2009).

Clinical and demographic information including age, gender, weight, height, ethnicity, duration of the operation and co-medication were collected.

During surgery, all patients had a standard general anesthesia using propofol and muscle relaxant for the induction and O_2_ and N_2_O, sevoflurane, fentanyl and muscle relaxant for the maintenance of anesthesia. They received morphine intravenously (IV) before extubation (0.05–0.1 mg/kg), followed by titration in the post-anesthesia care unit (PACU) until VAS ≤ 3 and acetaminophen 1 gr q6 IV for 48 h. PCA was started in PACU with 2 mg/10 min for 48 h with possibility to raise bolus to 3 mg/10 min if the VAS was ≥5.

Pain was self-rated by the patients using the item of “average pain” by the VAS during 24 h. Patients rated pain on a numeric scale, where 0 represents “no pain” and 10 “pain as bad as you can imagine” as recommended for use in clinical studies of pain. The pain scores were reported in integer number.

The cumulative 24 and 48 h doses of morphine were collected, including morphine administered peroperatively, in the PACU, and those administered by PCA.

Side effects commonly associated with morphine treatment were also assessed. Sedation was evaluated on a scale going from (0–4) (0 = Cooperative, orientated and tranquil; 1 = Responding to commands; 2 = Responding to tactile stimulation; 3 = Responding to painful stimulation; 4 = No response to stimulus), as well as nausea and vomiting from (0–2) (0 = Absence of nausea; 1 = Mild to moderate nausea; 2 = Severe nausea requiring antiemetic). Respiratory depression (respiratory rate less than 10/min) and bradycardia (pulse less than 45/min) were evaluated according to the qualitative criteria Presence/Absence (yes/no: Y/N). Anxiety was evaluated in the PACU using a 0 to 100 scale, where 0 represents “no anxiety” and 100 represents “very severe anxiety”.

### 3.2. Genotyping

DNA was extracted from blood cells using the QIAamp DNA Mini^®^ Blood (Qiamp DNA Mini kit cat nb: 51304, QIAGEN^®^, Hilden, Germany) as recommended by the manufacturer.

Genotyping for the two SNPs was performed using the Lightcycler^®^ 2.0 (Roche Diagnostics GmbH, Mannheim, Germany).

In summary, the reaction was carried out using 25 ng of DNA (10 ng/μL solution or 2.5 µL) in a final volume of 10 µL. The reaction mixture (10 μL) contained Fast Start Taq polymerase (10×), buffer and dNTPs, MgCl_2_ (10 mM); Lightcycler Fast Start DNA Master Hybridization Probes Kit^®^ catalogue no. 03 003 248 001, Roche Diagnostics GmbH, Mannheim, Germany), and 0.2 μL of each primer (20 mM) and fluorescent probes (anchor and sensor, 20 mM) (TIB Molbiol^®^, TIBMOLBIOL, Berlin, Germany). The samples were then loaded into composite plastic/glass capillaries (20 μL LC capillaries, Roche Diagnosis, catalogue no. 04 929 292 001, Roche Diagnostics GmbH, Mannheim, Germany), centrifuged, and placed in the LightCycler sample carousel.

Genotyping of *OPRM1* (rs1799971) and *ABCB1* (rs1045642) were performed according to previously published methods [38,39]. Primers and probes were synthesized using TIB MOLBIOL Syntheselabor GmbH, Berlin, Germany. The sequences were as follows: for *OPRM1* c.118A>G, primer forward 5'-GCTTGGAACCCGAAAAGT-3', primer reverse 5'-GTAGAGGGCCATGATCGTGA-3', probes CCCGGTTCCTGGGTCAACTTGTCC-FL and 640-CTTAGATGGCAACCTGTCCGACC-PH and for ABCB1 c.3435C>T, primer forward 5'-TGTTTTCAGCTGCTTGATGG-3', primer reverse 5'-AAGGCATGTATGTTGGCCTC-3', probes 640-GACAACAGCCGGGTGGTGTCA and GGAAGAGATCGTGAGGGCAG-PH.

Positive heterozygous and homozygous controls (defined by direct sequencing) and negative controls (water) were systematically included in experiments.

The genotyping was conducted on patients following their surgery. The genotyping was performed in the laboratory and none of the investigators, clinical care providers or observers of this study were aware about the genotyping results. Therefore, the genetic testing could not have biased the pain assessment process.

### 3.3. Data and Statistical Analysis

Clinical data are presented as mean ± standard deviation (SD). The statistical analysis was performed using a software program (SPSS for Windows version 16.0, SPSS Inc., Chicago, IL, USA). The alpha error was set at 0.05.

The formula for calculating sample size requirements was the one published by Tabachnick and Fidell [40] that takes into account the number of independent variables included in the model: *N* = 50 + 8 *m* (*m* is the number of independent variables); given that *m* = 5, at least 90 subjects have to be included in the present study.

Deviation from the Hardy-Weinberg equilibrium was tested using χ^2^ analysis with one degree of freedom.

To determine the genetic and non-genetic factors associated with the morphine doses at 24 h, the doses were dichotomized using the 50th percentile. In the initial stage, the univariate analyses of categorical and continuous variables were carried out using the χ^2^ independence tests and the Student’s *t*-test or the analysis of variance, respectively. A logistic regression model was carried out with one categorical dependent variable (doses of morphine) and the explanatory independent variables. Explanatory variables, that showed associations to the doses of morphine with *p* < 0.25 in univariate analyses, were candidates for the multivariate model according to the Enter method.

## 4. Conclusions

Our study adds to the evidence that age, weight, duration of the operation, *OPRM1* c.118A>G and *ABCB1* c.3435C>T genotypes influence postoperative morphine doses required by PCA for good pain relief during the 24 h after major painful surgeries.

It would be of interest in future studies to evaluate the added value of a preoperative genotype testing for acute pain management. Hence, if our results are confirmed on a larger cohort, it will emphasize the importance of individualizing analgesic therapy to optimize medical treatment, in terms of efficacy, safety and cost. A preoperative genotyping could determine acute pain management strategies. Rational doses of standard (morphine) or minor analgesics treatment might be sufficient for 118AA and 3435TT patients to have good analgesia, while patients with at least 118G or 3435C alleles (either 118AG or GG and 3435CC/CT) would be ideal candidates for a PCA-morphine based on a “multimodal” analgesia, combined with local regional or peripheral nerve blocks analgesia techniques. This could provide adequate pain relief as well as a reduction in the occurrence of analgesic side effects after major surgeries.

Ideally, in the future, it would be interesting to elaborate a guided dosing algorithm incorporating genotypes in addition to some clinical and demographic factors such as age, weight and duration of the operation. This algorithm would improve treatment outcomes of acute postoperative pain management strategies.

## Supplementary Materials

Supplementary materials can be found at http://www.mdpi.com/1422-0067/16/03/6298/s1.
