# Supplementary material for: Genotyping Test with Clinical Factors: Better Management of Acute Postoperative Pain?
Source: Int J Mol Sci. 2015 Mar 19;16(3):6298–311. doi: 10.3390/ijms16036298 (PMC4394533; doi:10.3390/ijms16036298)
Supplement: Supplementary file 1 [file ijms-16-06298-s001.pdf]

Supplementary Information

Table S1. Association between *OPRM1* SNP and sedation at 24 h (No statistical difference $p > 0.05$; Fisher Exact Test).

Gene	Genotype	Sedation at 24 h			Total
		**Cooperative, Orientated and Tranquil**	**Responding to Commands**	**Responding to Tactile Stimulation**	
OPRM1	AA	67 (79.8%)	8 (80.0%)	1 (100.0%)	76
	AG	15 (17.9%)	2 (20.0%)	0 (0.0%)	17
	GG	2 (2.4%)	0 (0.0%)	0 (0.0%)	2
Total		84 (100.0%)	10 (100.0%)	1 (100.0%)	95

Table S2. Association between *OPRM1* SNP and nausea at 24 h (No statistical difference $p > 0.05$; Fisher Exact Test).

Gene	Genotype	Sedation at 24 h			Total
		**Absence of Nausea**	**Mild to Moderate Nausea**	**Severe Nausea Requiring Antiemetic**	
OPRM1	AA	65 (79.3%)	8 (80.0%)	3 (100.0%)	76
	AG	15 (18.3%)	2 (20.0%)	0 (0%)	17
	GG	2 (2.4%)	0 (0.0%)	0 (0%)	2
Total		84 (100.0%)	10 (100.0%)	3 (100.0%)	95

Table S3. Association between *OPRM1* SNP and vomiting at 24 h (No statistical difference $p > 0.05$; Fisher Exact Test).

Gene	Genotype	Vomiting at 24 h		Total
		**Vomiting −**	**Vomiting +**	
OPRM1	AA	75 (79.8%)	1 (100.0%)	76
	AG	17 (18.1%)	0 (100.0%)	17
	GG	2 (2.1%)	0 (100.0%)	2
Total		94 (100.0%)	1 (100.0%)	95

Table S4. Association between *ABCB1* SNP *and* sedation at 24 h (No statistical difference $p > 0.05$; Fisher Exact Test).

Gene	Genotype	Sedation at 24 h			Total
		**Cooperative, Orientated and Tranquil**	**Responding to Commands**	**Responding to Tactile Stimulation**	
ABCB1	CC	30 (35.7%)	3 (30.0%)	0 (0%)	33
	CT	33 (39.3%)	4 (40.0%)	1 (100.0%)	38
	TT	21 (25.0%)	3 (30.0%)	0 (0%)	24
Total		84 (100.0%)	10 (100.0%)	1 (100.0%)	95

Table S5. Association between *ABCB1 SNP* and nausea at 24 h (No statistical difference $p > 0.05$; Fisher Exact Test).

Gene	Genotype	Sedation at 24 h			Total
		Absence of Nausea	Mild to Moderate Nausea	Severe Nausea Requiring Antiemetic	
ABCB1	CC	28 (34.1%)	3 (30.0%)	2 (66.7%)	33
	CT	34 (41.5%)	4 (40.0%)	0 (0.0%)	38
	TT	20 (24.4%)	3 (30.0%)	1 (33.3%)	24
Total		82 (100.0%)	10 (100.0%)	3 (100.0%)	95

Table S6. Association between *ABCB1* SNP and vomiting at 24 h (No statistical difference $p > 0.05$; Fisher Exact Test).

Gene	Genotype	Vomiting at 24 h		Total
		Vomiting −	Vomiting +	
ABCB1	CC	32 (34.0%)	1 (100.0%)	33
	CT	38 (40.4%)	0 (100.0%)	38
	TT	24 (25.5%)	0 (100.0%)	24
Total		94 (100.0%)	1 (100.0%)	95

Table S7. Association between age, weight, duration of the operation and sedation at 24 h (No statistical difference $p > 0.05$; Fisher Exact Test).

Characteristics of the Subjects	Sedation at 24 h	*N*	Mean	Std. Deviation	Sig.
Age	Cooperative, orientated and tranquil	84	51.88	13.956	0.164
	Responding to commands	10	43.50	13.591	
	Responding to tactile stimulation	1	58.00		
Weight	Cooperative, orientated and tranquil	84	75.40	14.842	0.360
	Responding to commands	10	74.90	14.012	
	Responding to tactile stimulation	1	94.00		
Duration of the operation	Cooperative, orientated and tranquil	80	213.62	115.531	0.244
	Responding to commands	9	171.11	66.978	
	Responding to tactile stimulation	1	360.00		

Table S8. Association between age, weight, duration of the operation and nausea at 24 h (No statistical difference $p > 0.05$; Fisher Exact Test).

Characteristics of the Subjects	Nausea at 24 h	*N*	Mean	Std. Deviation	Sig.
Age	Absence of nausea	82	50.04	14.019	0.215
	Mild to moderate nausea	10	56.50	13.159	
	Severe nausea requiring antiemetic	3	61.00	13.229	
Weight	Absence of nausea	82	75.77	15.371	0.382
	Mild to moderate nausea	10	72.00	10.593	
	Severe nausea requiring antiemetic	3	81.33	3.215	

Table S8. *Cont.*

Characteristics of the Subjects	Nausea at 24 h	*N*	Mean	Std. Deviation	Sig.
Duration of the operation	Absence of nausea	78	210.96	115.292	0.589
	Mild to moderate nausea	9	227.22	103.595	
	Severe nausea requiring antiemetic	3	163.33	66.583	

Table S9. Association between age, weight, duration of the operation and vomiting at 24 h (No statistical difference $p > 0.05$; Fisher Exact Test).

Characteristics of the Subjects	Vomiting	*N*	Mean	Std. Deviation	Sig.
Age	Vomiting −	94	51.01	14.097	0.841
	Vomiting +	1	56.00		
Weight	Vomiting −	94	75.51	14.805	0.756
	Vomiting +	1	79.00		
Duration of the operation	Vomiting −	89	210.67	113.145	0.562
	Vomiting +	1	240.00		

Table S10. Association between *OPRM1* SNP and VAS scores at rest and on movement (No statistical difference $p > 0.05$; Fisher Exact Test).

VAS at 24 h	Genotypes	*N*	Mean	Std. Deviation	Sig.
VAS rest	AA	76	0.96	1.321	0.982 Kruskal Wallis test
	AG	17	1.06	1.749	
	GG	2	1.00	1.414	
	Total	95	0.98	1.391	
VAS movement	AA	76	1.86	1.671	0.691 Kruskal Wallis test
	AG	17	2.29	2.085	
	GG	2	2.50	2.121	
	Total	95	1.95	1.747	

Table S11. Association between *ABCB1* SNP and VAS scores at rest and on movement (No statistical difference $p > 0.05$; Fisher Exact Test).

VAS at 24 h	Genotypes	*N*	Mean	Std. Deviation	*p*-Value
VAS rest	CC	33	0.91	1.444	0.724 Kruskal Wallis test
	CT	38	1.03	1.498	
	TT	24	1.00	1.180	
	Total	95	0.98	1.391	
VAS movement	CC	33	2.06	1.638	0.582 Kruskal Wallis test
	CT	38	2.03	1.924	
	TT	24	1.67	1.633	
	Total	95	1.95	1.747	

Table S12. Association between *age*, weight, duration of the operation and VAS scores at rest and on movement (No statistical difference $p > 0.05$; Fisher Exact Test).

VAS at 24 h		Age	Weight	Duration of the Operation
VAS rest	Pearson Correlation	−0.117	−0.026	0.225
	Sig. (2-tailed)	0.258	0.803	0.033 *
	N	95	95	90
VAS movement	Pearson Correlation	−0.120	−0.043	0.116
	Sig. (2-tailed)	0.248	0.681	0.276
	N	95	95	90

* Statistically significant result.
